# Artificial Intelligence in Healthcare: A Study of Physician Attitudes and Perceptions in Jeddah, Saudi Arabia

**DOI:** 10.7759/cureus.57256

**Published:** 2024-03-30

**Authors:** Mahmood Alkhatieb, Abeer A Subke

**Affiliations:** 1 Preventive Medicine Postgraduate Program, Saudi Ministry of Health, Jeddah, SAU

**Keywords:** perception, healthcare, attitudes, physicians, artificial intelligence

## Abstract

Background

Artificial intelligence (AI) in healthcare is rapidly advancing, reshaping diagnostic, prognostic, and operational tasks in healthcare institutions. The adoption of AI among physicians is varied, with concerns over job loss, medical errors, and lack of emotional intelligence. This study aimed to assess physicians’ attitudes and perceptions toward AI in clinical practice in Jeddah, Saudi Arabia, and the factors affecting these attitudes and perceptions.

Methodology

A cross-sectional study was conducted among physicians at two major hospitals in Jeddah. An in-person digital survey consisted of questions regarding demographic characteristics, attitudes toward clinical AI, and perceptions of AI’s impact on healthcare.

Results

Of the 205 participants, 76% agreed on the accuracy of AI systems, and 60% acknowledged their efficiency as a factor that could influence their willingness to use clinical AI. However, only 25.9% reported using these systems in the past year, with the majority, 74.1%, indicating they had never used them. Notably, there was a significant association between gender and attitude toward AI, with males being more likely to have a positive attitude (p = 0.01).

Conclusions

While the majority of participants recognized the potential benefits of AI in healthcare, its actual utilization was low. The findings suggest the need for increased AI-related training and education among physicians and the fostering of collaboration between computer scientists, engineers, and medical professionals to accelerate the development of clinically relevant AI tools.

## Introduction

Artificial intelligence (AI), including machine learning (ML) and deep learning, is used to perform cognitive tasks such as problem-solving and decision-making, which is particularly helpful in the healthcare sector [1]. It assists in diagnostics, prognosis, and operational functions in hospitals, showing promise in analyzing medical images as effectively as human specialists [2,3]. ML, a branch of AI, recognizes patterns in data to understand or predict based on them [1]. AI’s ability to analyze complex data can optimize healthcare processes and potentially reduce errors such as incorrect medication administration [2].

AI has improved hospital operations at institutions such as the University of Colorado Health and New York Presbyterian Hospital, particularly in surgical scheduling and reducing patient wait times [2]. Sharp Health Care in San Diego utilizes AI to forecast admissions and streamline workflows, reducing patient transfer times [2].

In Emergency Medicine, AI shows great potential, especially in clinical prediction and diagnostics, including predicting cancer outcomes, thus easing the workload on healthcare professionals [4]. AI has also been instrumental in diagnosing COVID-19 through CT and MRI scans [5]. It has demonstrated economic benefits by enhancing efficiency and productivity in healthcare. Specialties such as radiology, dermatology, and cardiology, which generate large volumes of structured data, are particularly suitable for AI applications [5].

AI in surgical care can analyze real-time operative data and patient variables to anticipate adverse events, aid in surgery planning, and predict postoperative complications. It can also consolidate global surgical experiences for insights into unique cases [6,7]. Despite its potential, AI’s lack of empathy poses a challenge [4]. However, surveys suggest physicians are likely to adopt AI as a tool to enhance their skills [4].

The US Food and Drug Administration has authorized 500+ medical AI applications [8]. Research highlights the necessity of physician involvement in AI tool development. A survey showed Canadian Emergency Physicians are keenly interested in AI for automated charting, clinical prediction, and patient triaging. Half of them already use AI tools, indicating acceptance. Radiologists find AI helpful for diagnostics and efficiency. AI techniques such as neural networks aid cardiologists in diagnostic imaging. AI influences everything from diagnostics to hospital operations [4,7].

Healthcare professionals, especially physicians, have a generally positive attitude toward AI, but concerns exist. These include potential job losses, severe errors, and a lack of emotional intelligence that AI cannot replicate. In a study among radiology residents in Jeddah, Saudi Arabia, 41.6% expected job positions to decrease due to AI [9]. Other concerns include data quality, biases in clinical data, and the“black box” nature of some AI techniques. Over-reliance on AI could also impact the doctor-patient relationship [4,7,8].

Under the Vision 2030 plan, Saudi Arabia prioritizes healthcare. The Saudi Data and Artificial Intelligence Authority focuses on data and AI to meet healthcare demands. Proper use of data and AI can help the sector face challenges and seize growth opportunities [10].

## Materials and methods

Study design and participants

We conducted an analytical cross-sectional study involving physicians at hospitals managed by the Ministry of Health in Jeddah, Saudi Arabia. The study was conducted at two of Jeddah’s largest hospitals, King Abdullah Medical Complex and King Abdulaziz Hospital, representing the city’s northern and southern areas. We included all physicians in these hospitals from all specialties except non-Saudi physicians.

We used a web-based sample size calculator from Raosoft, Inc., to determine our sample size. Given a 5% margin of error, a 95% confidence level, an estimated population size of 1,000, and a response distribution of 50%, the calculator suggested a sample size of 278 participants to ensure statistical reliability and validity. However, we received a total of 205 valid responses.

Data collection

We utilized an 18-question English-language questionnaire from a systematic review that employed a cross-sectional method to explore the same topic [11]. This questionnaire was split into the following three primary sections: participant demographic characteristics and experiences, attitudes toward clinical AI, and perceptions of AI’s impact on healthcare. The attitude score ranged from a minimum of 14 to a maximum of 70. Participants scoring 43 or above were deemed to have a positive attitude toward clinical AI. The researchers approached potential participants in the hospitals and extended an invitation to participate in the study. Those who consented to participate filled out the online questionnaire independently.

Data analysis

We performed statistical analyses using SPSS Software, version 29.0 (IBM Corp., Armonk, NY, USA). The Kolmogorov-Smirnov test was used to verify data normality, followed by descriptive statistics for data summarization. Categorical variables were expressed as proportions and numerical variables as median ± interquartile range. We used chi-square and Fisher-Freeman-Halton exact tests for univariate analysis of categorical outcomes. A p-value less than 0.05 was considered significant, and conclusions were drawn at a 95% confidence level.

Ethical considerations

We obtained research approvals from the Preventive Medicine Residency Program Ethics Committee and the Research and Studies Department at the Directorate of Health Affairs in Jeddah (approval number: A01804). Before collecting data, we received written informed consent from all participants, thoroughly explaining the study objectives beforehand. We also anonymized all data to ensure confidentiality and privacy.

## Results

Our study included a total of 205 participants with a diverse range of backgrounds. The median age of the participants was 30 years, with an interquartile range of 10 years, and the largest age group was those aged 30 years or less, which constituted 53.7% of the total. Regarding gender, a slight majority of the participants were female, accounting for 56.1% of the sample. Regarding education, most participants held a bachelor’s degree, accounting for 61% of the cohort. Concerning work experience, a significant portion of the participants, 57.1% to be precise, had five years of work experience or less, indicating a relatively young workforce. The participants’ professional fields were almost evenly divided between the medical and surgical specialties. Specifically, 64.9% were from the medical field, and the remaining 35.1% were from surgical specialties (Table 1).

**Table 1:** Demographic characteristics.

N = 205		N	%
Age	≤30 years	110	53.7%
	31–40 years	72	35.1%
	41–50 years	18	8.8%
	>50	5	2.4%
Gender	Male	90	43.9%
	Female	115	56.1%
Education	Bachelor’s degree	125	61.0%
	Master’s or higher degree	80	39.0%
Professional title	General physician	61	29.8%
	Resident physician	62	30.2%
	Specialist	31	15.1%
	Consultant	51	24.9%
Work experience	≤5 years	117	57.1%
	6–10 years	40	19.5%
	11–15 years	28	13.7%
	>15 years	20	9.8%
Specialty	Medical	133	64.9%
	Surgical	72	35.1%
Hospital	King Abdulaziz Hospital	88	42.9%
	King Abdullah Medical Complex	117	57.1%

Regarding their engagement with clinical AI systems, only a small percentage, precisely 25.9% of the survey participants, reported having utilized these systems in their professional practice over the past year. The frequency of usage was also notably low, with the majority, 74.1%, of the respondents disclosing that they had never used these systems. Meanwhile, a small fraction, just 2%, reported using these systems daily. Furthermore, 11.7% of the participants reported experiencing errors or accidents while working with these systems. Regarding patient attitudes, most participants reported that the patient’s views on using decision-support clinical AI systems were unclear, with a significant majority of 62% indicating this (Table 2).

**Table 2:** Practice experience of clinical artificial intelligence (AI).

N = 205		N	%
In the past year, how often have you used decision-support clinical AI systems in practice?	Never	152	74.1%
	Only once a year	20	9.8%
	At least once every six months	16	7.8%
	At least once a month	5	2.4%
	At least once a week	8	3.9%
	Everyday	4	2.0%
Have any errors or accidents occurred while working with decision-support clinical AI systems?	Never worked on decision-support clinical AI systems	152	74.1%
	Yes	24	11.7%
	No	29	14.1%
What are patients’ attitudes toward the use of decision-support clinical AI systems?	Oppose	8	3.9%
	Neutral	52	25.4%
	Support	18	8.8%
	Unclear	127	62.0%

We investigated the participants’ attitudes toward AI and its association with various factors. Notably, there was a significant association between gender and attitude toward AI (p = 0.01), with males being more likely to have a positive attitude. However, no significant association was found with other factors such as age, education, professional title, work experience, specialty, or hospital (Table 3).

**Table 3:** Associated factors with participants’ attitudes. Chi-square and Fisher-Freeman-Halton exact tests. *: p-value <0.05.

N = 205		Attitude				
		Negative		Positive		
		N	%	N	%	P-value
Age	≤30 years	70	57.9%	40	47.6%	0.492
	31–40 years	39	32.2%	33	39.3%	
	41–50 years	9	7.4%	9	10.7%	
	>50 years	3	2.5%	2	2.4%	
Gender	Male	44	36.4%	46	54.8%	0.01
	Female	77	63.6%	38	45.2%	
Education	Bachelor’s degree	77	63.6%	48	57.1%	0.384
	Master’s or higher degree	44	36.4%	36	42.9%	
Professional title	General physician	43	35.5%	18	21.4%	0.132
	Resident physician	35	28.9%	27	32.1%	
	Specialist	18	14.9%	13	15.5%	
	Consultant	25	20.7%	26	31.0%	
Work experience	≤5 years	78	64.5%	39	46.4%	0.083
	6–10 years	20	16.5%	20	23.8%	
	11–15 years	13	10.7%	15	17.9%	
	>15 years	10	8.3%	10	11.9%	
Specialty	Medical	83	68.6%	50	59.5%	0.234
	Surgical	38	31.4%	34	40.5%	
Hospital	King Abdulaziz Hospital	49	40.5%	39	46.4%	0.473
	King Abdullah Medical Complex	72	59.5%	45	53.6%	

**Figure 1:**
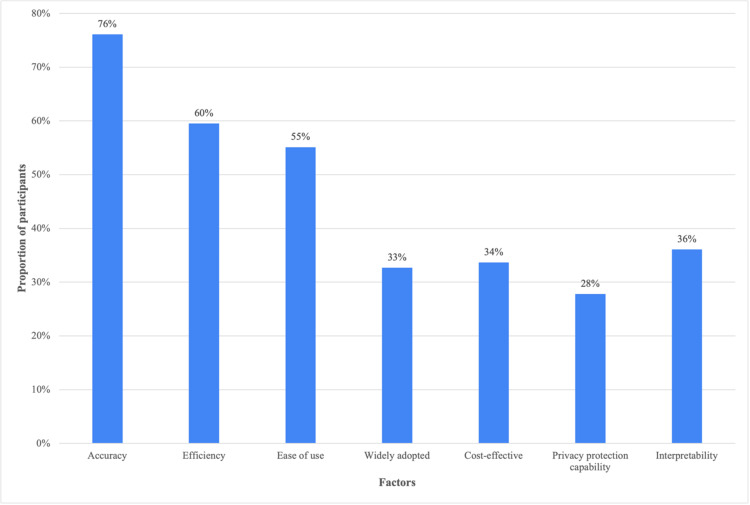
Factors associated with willingness to use clinical artificial intelligence (AI).

When analyzing participants’ perceptions of clinical AI attributes, 76% agreed that system accuracy and 60% agreed that efficiency could influence their willingness to use clinical AI. Overall, 55% of the respondents acknowledged ease of use, while 33% considered the broad adoption of AI as an attribute. Cost-effectiveness and privacy protection capability were less recognized, with only 34% and 28% agreement, respectively. Additionally, only 36% of the participants confirmed the interpretability of AI (Figure 1).

**Figure 2:**
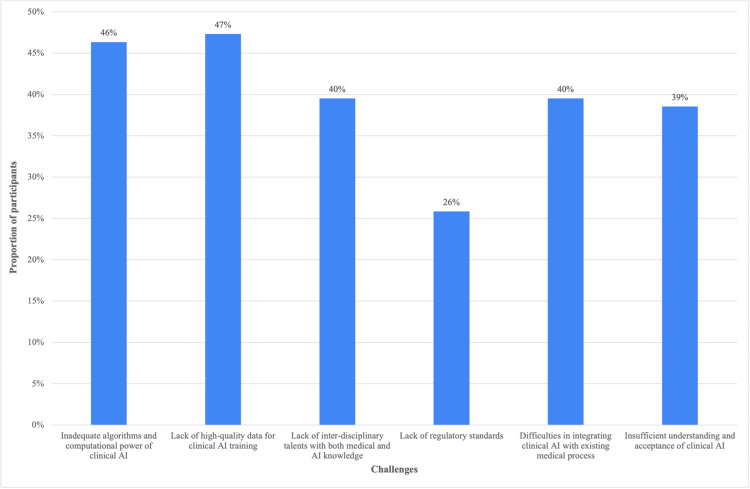
Challenges to be overcome in the development and implementation of clinical artificial intelligence (AI).

Regarding challenges faced in applying clinical AI, many respondents cited the inadequacy of algorithms and computational power (46%) and lack of high-quality data for AI training (47%) as significant hurdles. The lack of interdisciplinary talents with medical and AI knowledge was pointed out by 40% of the respondents. Regulatory standards and difficulties integrating AI with existing medical processes were also mentioned as obstacles by 26% and 40% of the participants, respectively. Interestingly, 39% of the respondents indicated insufficient understanding and acceptance of clinical AI as a challenge (Figure 2).

**Figure 3:**
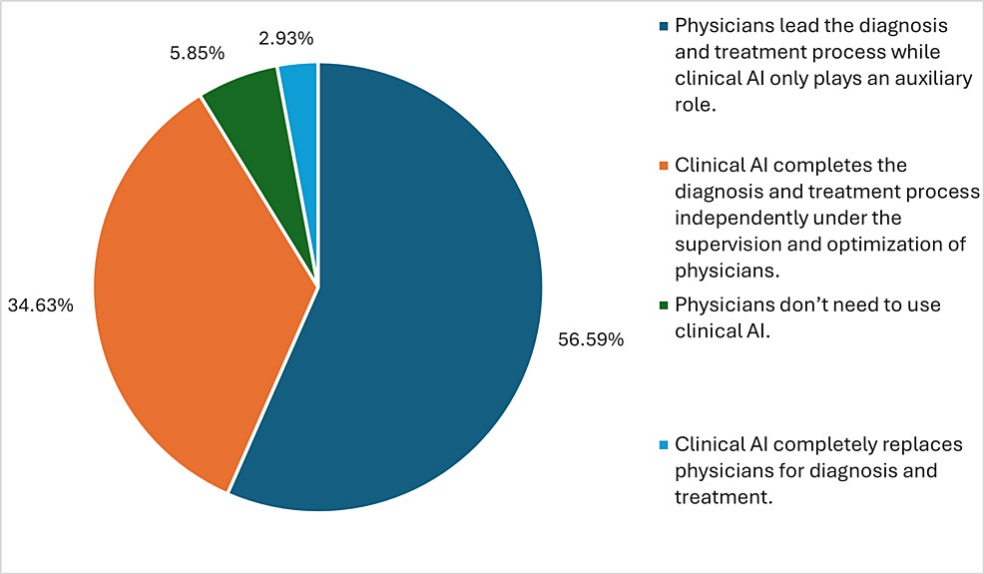
Perceived relationship between physicians and clinical artificial intelligence (AI).

Finally, when asked about the role of clinical AI in diagnosis and treatment, most participants (56.6%) agreed that physicians should lead the process while AI only plays an auxiliary role. A significant portion (34.6%) believed that AI could independently complete the diagnosis and treatment process under the supervision and optimization of physicians. Only a small fraction of the respondents (2.9%) thought that AI could completely replace physicians for diagnosis and treatment. Conversely, 5.9% of the participants believed that physicians do not need to use clinical AI (Figure 3).

## Discussion

Our study investigated the knowledge, attitude, and perception of physicians in Jeddah, Saudi Arabia, regarding clinical AI systems. A key finding of our study is the discrepancy between physician’s recognition of the potential benefits of AI and its actual utilization. While 25.9% reported using AI in the past year, 40.1% had a positive attitude toward its usage, while the majority, 74.1%, had never used these systems. These findings align with multiple studies across various regions. For instance, a German study reported that only 42.9% of physicians had a positive or very positive attitude toward AI in medicine, despite most believing it would play a future role [12,13]. Similarly, a US survey found that while Americans anticipated benefits from AI in medicine, their willingness to adopt specific applications varied [14]. These findings suggest a global trend where a positive perception of AI does not necessarily translate to widespread implementation.

Several factors could contribute to this gap. One possibility, highlighted in our study and echoed in the study by Orlova et al., is the lack of training and education on AI applications among physicians [15]. Many may feel unequipped to use these systems effectively or harbor concerns about their reliability and transparency. Additionally, our study points to a significant portion of respondents, 74.1%, having no previous experience with decision-support clinical AI systems. This suggests a potential need for increased awareness of the specific types of AI technologies available and how they can integrate into existing workflows.

Our findings of gender disparity, with male physicians exhibiting a more positive view of AI, align with the literature [12]. Further exploration is warranted to understand the reasons behind this difference. Other studies did not explore gender differences, highlighting a gap in the current body of research [15,16]. Future investigations could explore whether this disparity is related to broader societal attitudes toward technology among different genders or stems from variations in the perceived technical complexity of AI. Additional research could also examine whether gender-specific training programs might help to level up the playing field regarding AI adoption among physicians.

A significant portion of our participants, 46%, highlighted limitations in algorithms and computational power as a major hurdle to AI implementation. This aligns with findings from other studies [15,17]. Additionally, 47% of our respondents cited a lack of high-quality data for AI training, which was also a concern in the study by Chen et al. [18]. These findings underscore the crucial role of robust data collection and standardization practices in developing reliable AI algorithms for healthcare applications. Furthermore, 40% of our participants identified a lack of interdisciplinary talent with both medical and AI knowledge as a challenge. This highlights the importance of fostering collaboration between computer scientists, engineers, and medical professionals to bridge the knowledge gap and accelerate the development of clinically relevant AI tools.

Our study also sheds light on physician preferences regarding the role of AI in diagnosis and treatment. Most participants, 56.6%, agreed that physicians should lead decision-making, with AI as a supportive tool. This aligns with research from various regions, including Europe, Korea, and the United States [14-16]. For instance, a study in the Netherlands found that physician acceptance of AI-powered care pathways was significantly influenced by the belief that AI would enhance medical performance [19,20]. These findings suggest a global consensus among physicians that AI should be a collaborative tool, augmenting their expertise rather than replacing it. Future research could investigate how best to design human-AI interfaces that foster effective collaboration and optimize workflow within clinical teams.

While our findings on physician attitudes are generally consistent with international research, it is essential to acknowledge potential differences specific to the Saudi Arabian context. For instance, the study by Barakat et al. reported that a significant portion of Saudi ophthalmologists believed that AI would decrease the physician workforce [17]. Our study did not explicitly explore this concern, but future research could delve deeper into physician anxieties about job security in the context of AI adoption.

Additionally, cultural factors may influence attitudes towards AI. Studies have shown that individuals from collectivistic cultures, which emphasize group harmony and social norms, may be more receptive to technologies that support collaboration and shared decision-making [13,20]. As Saudi Arabia scores relatively high on collectivism scales, this could partially explain the preference for physician-led decision-making with AI as a supportive tool. Further research is needed to explore the influence of cultural backgrounds on attitudes towards AI adoption in healthcare settings across various countries.

Limitations and future directions

A limitation of our study is the sample size and the fact that we only included physicians from two representative hospitals in Jeddah. Future research could involve a larger, more geographically diverse sample to enhance generalizability. Additionally, qualitative research methods, such as interviews, could provide deeper insights into physician’s perceptions and concerns regarding AI in healthcare. Studies could explore how cultural backgrounds, practice settings, and areas of specialization influence attitudes toward AI adoption.

For instance, future research could investigate physicians’ perspectives on the ethical implications of AI in medicine. This could include concerns about bias in algorithms, data privacy and security, and the potential for over-reliance on AI at the expense of clinical judgment. Furthermore, studies could explore the broader impact of AI on the physician-patient relationship.

## Conclusions

Our study indicates that while there is a positive attitude toward AI among physicians in Jeddah, Saudi Arabia, its adoption in clinical practice is still limited. These findings highlight the need for further education and training to increase physicians’ understanding and utilization of AI. Additionally, addressing technical challenges and data quality concerns is crucial to promote the successful integration of AI into healthcare practices. Our study also underscores the desire of physicians to use AI as a supportive tool rather than a replacement, emphasizing the importance of maintaining the human element in patient care. Further research is needed to explore the cultural, ethical, and practical implications of AI adoption in healthcare settings. It is clear that AI has the potential to enhance healthcare delivery significantly, but it needs to be carefully managed to ensure it is used effectively and ethically.
